# REP1 deficiency causes systemic dysfunction of lipid metabolism and oxidative stress in choroideremia

**DOI:** 10.1172/jci.insight.146934

**Published:** 2021-05-10

**Authors:** Dulce Lima Cunha, Rose Richardson, Dhani Tracey-White, Alessandro Abbouda, Andreas Mitsios, Verena Horneffer-van der Sluis, Panteleimon Takis, Nicholas Owen, Jane Skinner, Ailsa A. Welch, Mariya Moosajee

**Affiliations:** 1 Department of Development, Ageing and Disease, UCL Institute of Ophthalmology, London, United Kingdom.; 2 Department of Genetics, Moorfields Eye Hospital NHS Foundation Trust, London, United Kingdom.; 3 MRC-NIHR National Phenome Centre, Department of Surgery and Cancer, Imperial College London, London, United Kingdom.; 4 Department of Public Health & Primary Care, Norwich Medical School, Norfolk, United Kingdom.; 5 Department of Ophthalmology, Great Ormond Street Hospital for Children NHS Foundation Trust, London, United Kingdom.; 6 The Francis Crick Institute, London, United Kingdom.

**Keywords:** Metabolism, Ophthalmology, Genetic diseases

## Abstract

Choroideremia (CHM) is an X-linked recessive chorioretinal dystrophy caused by mutations in *CHM*, encoding for Rab escort protein 1 (REP1). Loss of functional REP1 leads to the accumulation of unprenylated Rab proteins and defective intracellular protein trafficking, the putative cause for photoreceptor, retinal pigment epithelium (RPE), and choroidal degeneration. *CHM* is ubiquitously expressed, but adequate prenylation is considered to be achieved, outside the retina, through the isoform REP2. Recently, the possibility of systemic features in CHM has been debated; therefore, in this study, whole metabolomic analysis of plasma samples from 25 CHM patients versus age- and sex-matched controls was performed. Results showed plasma alterations in oxidative stress–related metabolites, coupled with alterations in tryptophan metabolism, leading to significantly raised serotonin levels. Lipid metabolism was disrupted with decreased branched fatty acids and acylcarnitines, suggestive of dysfunctional lipid oxidation, as well as imbalances of several sphingolipids and glycerophospholipids. Targeted lipidomics of the *chm^ru848^* zebrafish provided further evidence for dysfunction, with the use of fenofibrate over simvastatin circumventing the prenylation pathway to improve the lipid profile and increase survival. This study provides strong evidence for systemic manifestations of CHM and proposes potentially novel pathomechanisms and targets for therapeutic consideration.

## Graphical abstract

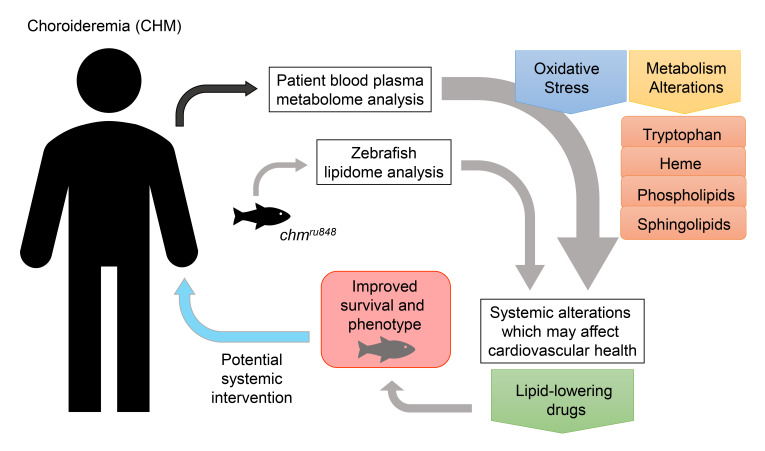

## Introduction

Choroideremia (CHM, OMIM 303100) is a chorioretinal dystrophy, with an incidence of 1 in 50,000–100,000, characterized by the progressive degeneration of photoreceptors (PR), retinal pigment epithelium (RPE), and choroid (1, 2). Affected male patients typically suffer from nyctalopia in the first decade of life that progresses to severe peripheral field loss with complete blindness in late adulthood, with no reports of associated systemic features (3). CHM is an X-linked recessive, monogenic disorder caused by mutations in the *CHM* gene (OMIM 303390). *CHM* encodes Rab escort protein 1 (REP1), an essential component of the catalytic Rab geranyl-geranyl transferase II (GGTase II) complex, which is essential for prenylation of Rab GTPase proteins (4, 5). Protein prenylation is a type of posttranslational lipid modification, which involves the covalent addition of either farnesyl- or GG-pyrophosphate (FPP or GGPP) to proteins via 3 different prenyltransferases: farnesyltransferase (FTase) and GGTase I and II (4). REP1 and its isoform REP2 recruit newly synthesized Rab GTPase proteins and present them to GGTase II, forming a tight catalytic complex in which 2 GGPP are transferred onto the C terminus. After prenylation, REP1/2 escorts the Rabs to their respective target membranes (6).

Since *CHM* is ubiquitously expressed, the possibility of systemic manifestations has long been considered but remains unproven. It is accepted that REP2 compensates for the REP1 deficiency, providing adequate prenylation of Rab proteins in all extraretinal tissues (7). Lack of this compensatory mechanism in the retina, due to preferential binding affinity of Rab27a, -6, -8, and -11 to REP1, is thought to lead to the accumulation of these unprenylated Rabs, resulting in an isolated ocular phenotype (5). However, an online self-reporting survey of 190 individuals — CHM male patients (*n* = 117), female carriers (*n* = 53), and unaffected males (*n* = 20) — undertaken by Zhou et al. suggested a higher prevalence of diabetes, high cholesterol, and hyperglycemia in CHM male patients, but these differences were not significant after age adjustment (8). They suggested that hydroxy-3-methyl-glutaryl-CoA (HMG-CoA) reductase inhibitors (also known as statins) for the treatment of hypercholesterolemia may have a negative effect on the visual function of CHM patients (8). GGPP and FPP are both isoprenoids produced through the mevalonate pathway, which is the main pathway for cholesterol synthesis and the target of these drugs (9). It has recently been reported that statins lead to lower pools of both isoprenoids necessary for GGTase activity and consequent inhibition of both farnesyl and GG prenylation (10–13).

Previously, analysis of lipid contents from blood samples of 5 CHM patients uncovered systemic fatty acid (FA) abnormalities in both plasma and RBCs. Specifically, lower levels of eicosenoic acid (C20:1[n-9]), erucic acid (C22:1[n-9]), and docosadienoic acid (C22:2[n-6]) were found in the plasma, with elevation of tridecaenoic acid (C13:1), myristolenic acid (C14:2), and octacosanoic acid (C28:0). RBCs revealed increased levels of capric acid (C10:0), nervonic acid (C24:1[n-9]), and plasmalogen derivative dimethylacetal acid (16:0), as well as a decrease in eicosenoic acid (C20:1[n-9]) (14). A follow-up report refuted these findings, stating that no lipid abnormalities were detected in the plasma of 9 CHM patients, nor could crystal deposits be detected after transmission and scanning electron microscopy analyses of WBCs and RBCs, respectively (15).

Herein, we performed whole metabolomic profiling of blood plasma from 25 CHM male patients and 25 age- and sex-matched controls to identify metabolic alterations in CHM and to gain insights into any systemic involvement. Several pathways were significantly altered in the disease cohort, including sphingolipid signal transduction, oxidative stress, and serotonin production.

Zebrafish have been acknowledged as a valuable model for studying metabolism and metabolic diseases (16, 17). Accordingly, targeted lipidomics analysis of the CHM zebrafish model *chm^ru848^*, with a C>T nonsense variant (p.[Gln32*]) in *chm* exon 2 on chromosome 21, confirmed lipid and sphingolipid alterations found in humans. Furthermore, we also undertook survival studies and lipidomic analysis of *chm^ru848^* embryos treated with simvastatin or fenofibrate (a fibric acid derivative mediated via activation of PPARα) to explore if prenylation is affected by statins. Zebrafish possess only 1 rep isoform, which leads to retinal degeneration from 4.5 days postfertilization (dpf) and multisystemic disease resulting in embryonic lethality by 5–6 dpf (18, 19).

This study discovers the metabolomic signature in CHM and identifies putative disease biomarkers, which may be critical to the future development of disease-modifying or preventative therapies.

## Results

### Patient description.

Detailed clinical and genetic evaluation of 25 male CHM patients is included in Supplemental Table 1 (supplemental material available online with this article; https://doi.org/10.1172/jci.insight.146934DS1). Mean ± SD age of CHM patients at the time of blood collection was 40.6 ± 11.4 years (range 20–63 years), with no significant difference with the control group (40.7 ± 12.3 years, *P* = 0.938). Analysis of the food frequency questionnaire (FFQ) revealed no significant dietary differences between disease and control groups with regard to average consumption of food and drink over the past 12 months (Supplemental Table 2).

### Global metabolite differences between CHM patients and controls.

**Figure 1:**
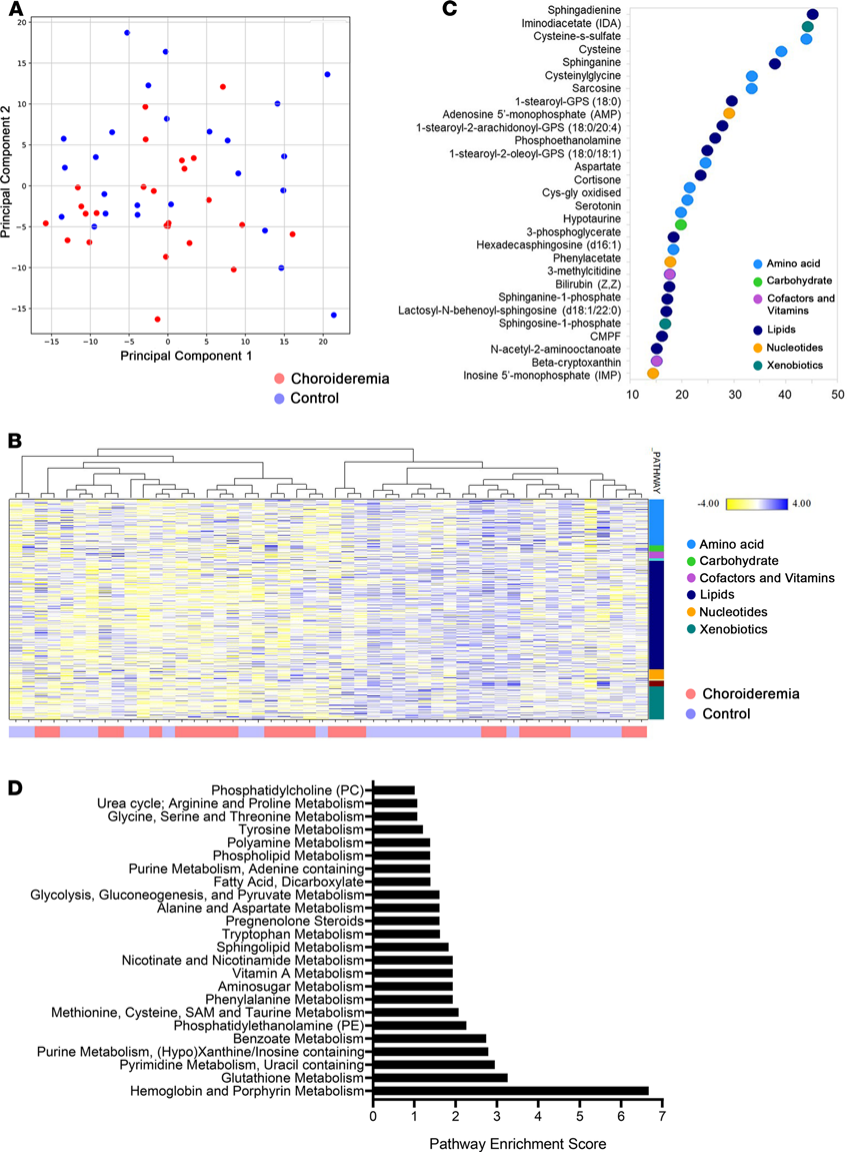
Global metabolomic analysis of choroideremia (CHM) patients versus age- and sex-matched controls. (**A**) Principal component analysis (PCA). Control and CHM samples are represented as blue and red circles, respectively (*n* = 25 each group). (**B**) Cluster analysis of control and CHM samples show no clear separation between groups. (**C**) Top 30 metabolites detected by random forest analysis based on importance to group separation. (**D**) Pathway analysis calculated using MetaboLync pathway analysis (MPA) software. Pathways with MPA score higher than 1 were considered.

In total, 817 compounds of known identity were detected in the blood plasma matrix of CHM patients and controls. Principal component analysis (PCA) of CHM patient and healthy control samples revealed largely overlapping grouping with no clear distinction between study groups (Figure 1A). Consistently, hierarchical cluster analysis of the data set revealed the same trend, with interdigitated sample clustering for healthy control and CHM patient samples (Figure 1B). Welch’s 2-sample *t* test (2 tailed) was used to identify compounds that differed significantly between CHM and healthy control study groups, with 85 named compounds achieving statistical significance (*P* ≤ 0.05) and a further 48 approaching significance (0.05 < *P* < 0.10).

### Individual biochemicals can differentiate between CHM patients and control study groups.

Random forest (RF) analysis indicated high probability that individual metabolites can distinguish between study groups, with a predictive accuracy of 86%. The top 30 metabolites based on distinguishing CHM from control groups are represented in Figure 1C and in more detail in Supplemental Table 3. These include several sphingolipid signal transducers (sphingosine, sphingadienine, sphinganine, hexadecasphingosine [d16:10], sphinganine 1-phosphate, and sphingosine 1-phosphate [S1P]); stearoyl-glycerophosphoserine (GPS) moieties for both lysolipids (e.g., 1-stearoyl-GPS [18:0]); and phosphatidylserine (PS) derivatives (e.g., 1-stearoyl-2-arachidonoyl-GPS [18:0/20:4] and 1-stearoyl-2-oleoyl-GPS [18:0/18:1]). Additionally, several metabolites in the cysteine pathway (cysteine s-sulfate, cysteine, cysteinylglycine, cys-gly oxidized) were highlighted (Figure 1C).

Pathway set enrichment analysis to elucidate the metabolic pathways affected between CHM patients and controls revealed significant perturbation of multiple networks, including oxidative stress, tryptophan metabolism, hemoglobin metabolism, and sphingolipid and lipid metabolisms (Figure 1D).

### CHM patients exhibit evidence of increased oxidative stress.

**Figure 2:**
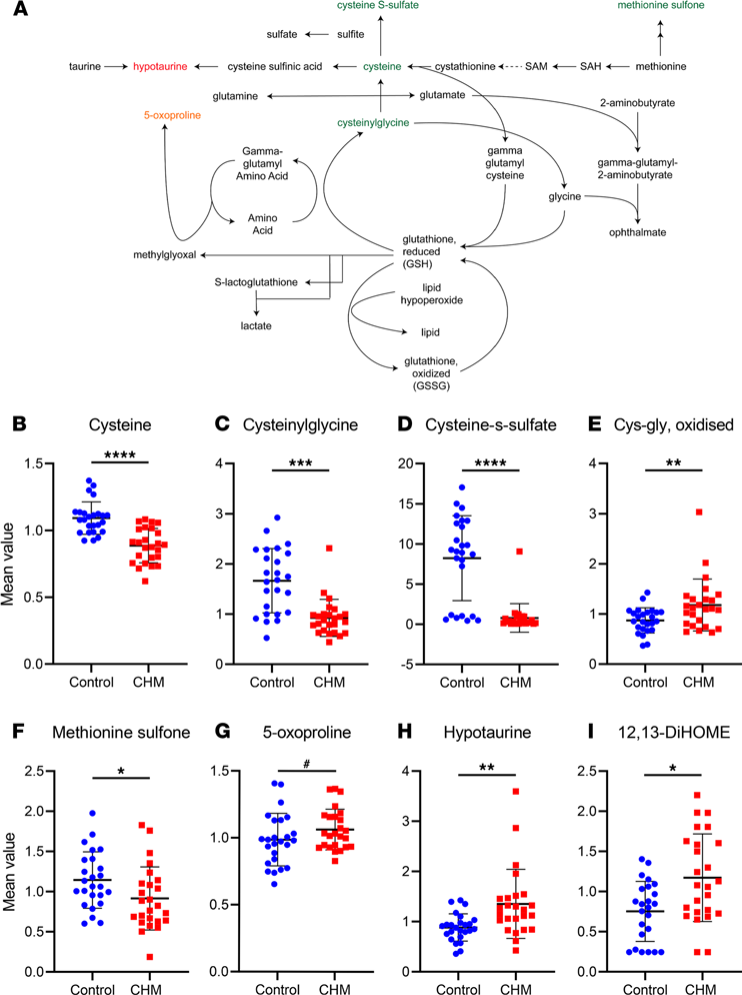
CHM patients exhibit evidence of increased oxidative stress. (**A**) Schematic of the glutathione metabolism pathway, where several compounds were found to be increased (red) or decreased (green) in CHM patients compared with controls. Metabolites trending to significance (0.05 < *P* ≤ 0.1) are represented in a lighter shade to distinguish from those significantly altered (*P* ≤ 0.05). (**B**–**I**) Scatter dot plots of specific metabolites indicating the mean ± SD levels in CHM patient samples (red) and control samples (blue) (*n* = 25). *P* value was determined using matched pair *t* tests. ^#^0.05 < *P* ≤ 0.1, *P ≤ 0.05, **P ≤ 0.01, ***P ≤ 0.001, ****P ≤ 0.0001.

**Figure 3:**
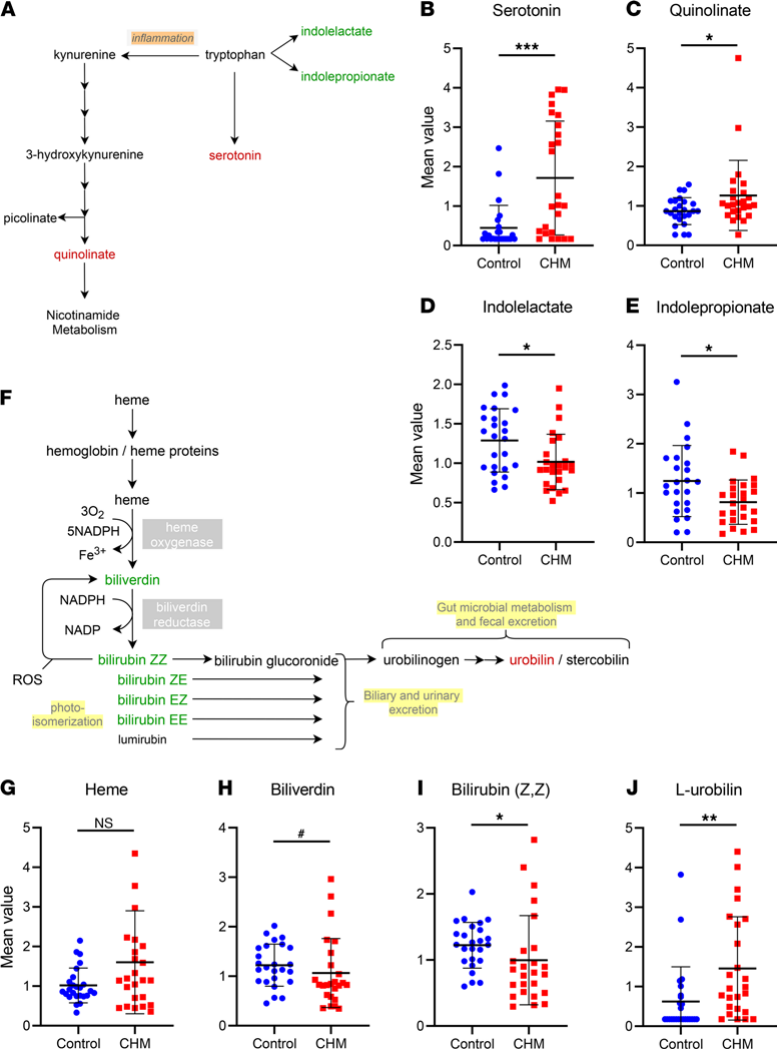
Alterations in tryptophan and hemoglobin metabolism pathways in CHM patients. (**A**) Pathway schematics and altered metabolites in tryptophan metabolism with decreased metabolites in green and increased in red. (**B**–**E**) Scatter dot plots of altered metabolites showing control (blue) and CHM (red) groups with mean ± SD (*n* = 25). (**F**) Schematic representation in the hemoglobin/heme metabolism pathway with decreased metabolites shown in green and increased metabolites in red. (**G**–**J**) Scatter dot plots of altered metabolites showing control (blue) and CHM (red) groups with mean ± SD (*n* = 25). *P* value was determined using matched pair *t* tests. *^#^*0.05 < *P* ≤ 0.1, *P ≤ 0.05, **P ≤ 0.01, ***P ≤ 0.001.

We observed mixed perturbations in the cysteine pathway (Figure 2A), such as loss of cysteine (fold change [FC] 0.81, *P* < 0.001) (Figure 2B) and associated dipeptide cysteinylglycine (FC 0.56, *P* < 0.001) (Figure 2C), as well as methionine sulfone (FC 0.80, *P* < 0.05) (Figure 2F), which combined may indicate an increased demand for the antioxidant glutathione. An elevation of oxidative stress marker cys-gly oxidized (FC 1.35, *P* < 0.01) (Figure 2E) and increase of lipid oxidation marker 12,13-DiHOME (FC 1.31, *P* < 0.05) (Figure 2I) were also observed, coupled with an accumulation of hypotaurine (FC 1.53, *P* < 0.01) (Figure 2H) and a trend increase in 5-oxoproline (FC 1.08, *P* < 0.1) (Figure 2G). Several other known antioxidants were found in significantly lower levels in CHM samples, like bilirubin (FC 0.80, *P* < 0.05) (Figure 3I), indolepropionate (FC 0.40, *P* < 0.05) (Figure 3E), β-cryptoxanthin (provitamin A) (FC 0.69, *P* < 0.05), urate (FC 0.87, *P* < 0.05), and suspected antioxidant 3-(3-hydroxyphenyl)propionate (FC 0.42, *P* < 0.005) (data not shown). Cysteine-s-sulfate is an incompletely characterized metabolite generated by reaction of cysteine and inorganic sulfite, and it was greatly reduced in CHM plasma (FC 0.10, *P* < 0.001) (Figure 2D). This likely relates to increased sulfite oxidase (SO) activity, which catalyzes the oxidation of sulfite to sulfate, the potentiation of which may divert from cysteine-s-sulfate production (Figure 2A). Combined, these observations seem to point to a deficient management of oxidative stress in CHM patients, possibly through deregulation of gluthathione metabolism, although glutathione levels are not usually detected in plasma.

### CHM patients display alterations in tryptophan metabolism.

The tryptophan metabolism pathway was enriched in this study, with a pathway enrichment score of 1.62, where a score greater than 1 indicated that the pathway contained a higher number of experimentally regulated compounds relative to the overall study in CHM patients relative to controls (Figure 1D and Figure 3A), with serotonin levels being strikingly elevated in CHM patients (FC 3.82, *P* < 0.001) (Figure 3B). Serotonin is an important monoaminergic neurotransmitter that regulates stress response, sleep, and behavior, among other body functions, and this increase indicates that tryptophan metabolism appears to be strongly shifted toward monoamine production. This observation is consistent with lower levels of alternative tryptophan catabolic pathways, involving microbiome-related indolelactate (FC 0.79, *P* < 0.05) and indolepropionate (FC 0.40, *P* < 0.05) in CHM patients (Figure 3, D and E). Also, there was no significant difference in nicotinamide levels between CHM and control samples (FC 1.02, *P* = 0.96) (not shown), although higher levels of quinolate (FC 1.45, *P* < 0.05) were detected (Figure 3C).

### Defects in cytochrome activity.

Hemoglobin synthesis and porphyrin metabolism pathway showed a high pathway enrichment score of 6.67 in this study (Figure 1D). Although only 6 compounds were analyzed overall (Figure 3F), we observed significantly lower levels of bilirubin isomers (Z, Z) (FC 0.82, *P* < 0.05) (Figure 3I), (E, Z or Z,E) (FC 0.82, *P* < 0.05) and (E, E) (FC 0.79, *P* < 0.05) (not shown), coupled to a trending reduction of its precursor biliverdin (FC 0.87, *P* < 0.1) in CHM samples (Figure 3H). In contrast, the product of bilirubin reduction, L-urobilin, was detected in higher levels (FC 2.53, *P* < 0.01) (Figure 3J). However, levels of heme were not significantly increased (FC 1.58, *P* > 0.1) (Figure 3G), suggesting that alterations occur downstream in the pathway.

While altered levels of bilirubin and urobilin could indicate increased heme breakdown in CHM patients, broader evidence may indicate lower liver cytochrome P450 activity. Cytochrome P450s metabolize several methylxanthines, which largely trend lower in CHM individuals, such as 3, 7-dimethylurate (FC 0.62, *P* < 0.05), 3-methylxanthine (FC 0.59, *P* < 0.1), caffeic acid sulfate (FC 0.61, *P* < 0.1), 7-methylurate (FC 0.48, *P* < 0.1), theobromine (FC 0.63, *P* < 0.1), and 7-methylxanthine (FC 0.60, *P* < 0.1) (data not shown). Additionally, cytochrome P450s also catalyze steroid biosynthesis, and a trending decrease in several steroid hormones and related metabolites in CHM patients was observed (Supplemental Figure 1), which further supports the hypothesis of impaired cytochrome activity in CHM patients.

### CHM patients exhibit disruption in sphingolipid metabolism.

**Figure 4:**
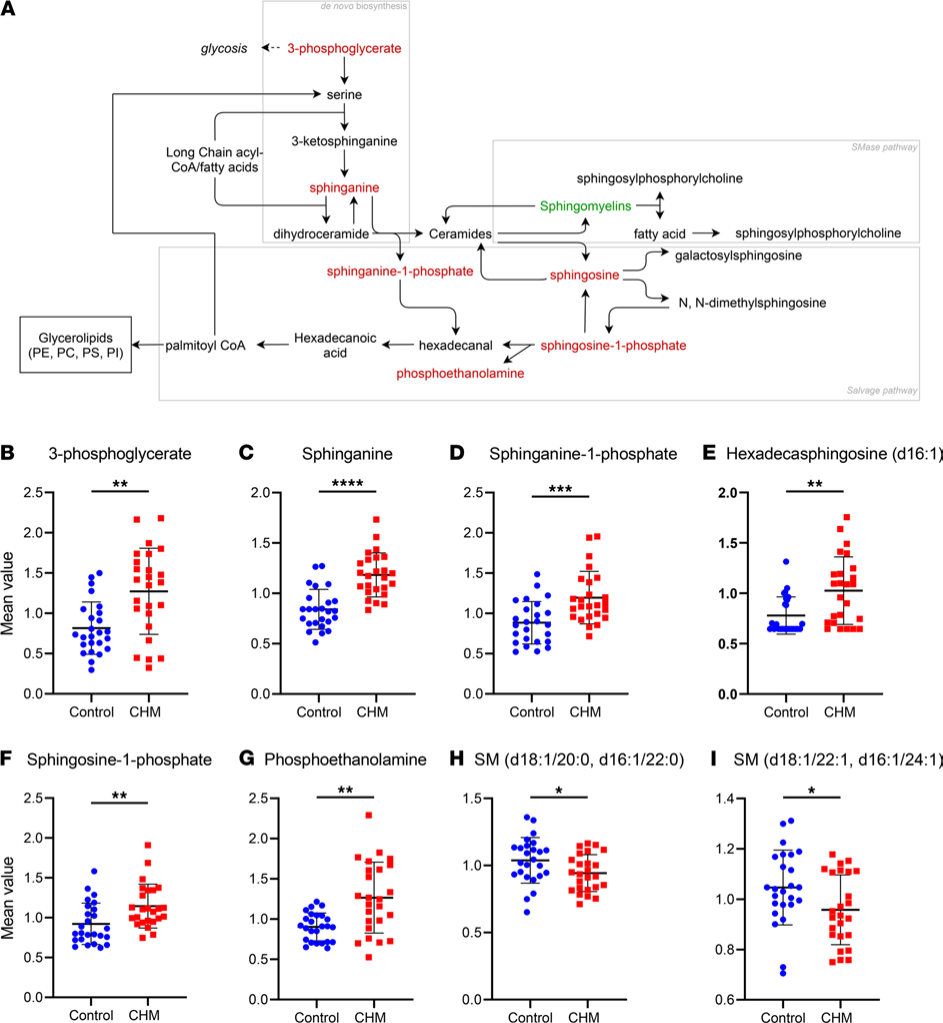
Disturbance of sphingolipid metabolism in CHM patients. (**A**) General sphingolipid metabolism pathway with compounds differentially detected in CHM patients highlighted in red (increased) or green (decreased) compared with control levels. (**B**–**I**) Scatter dot plots of key metabolite levels in both control (blue) and choroideremia (red) plasma samples. Lines indicate mean ± SD (*n* = 25). *P* value was determined using matched pair *t* tests. *P ≤ 0.05, **P ≤ 0.01, ***P ≤ 0.001, ****P ≤ 0.0001. SM, sphingomyelin.

Deeper analysis of the compounds identified by RF analysis revealed broader perturbation of the sphingolipid pathways in CHM patients (Figure 4). The sphingolipid pathway generates the bioactive lipid metabolite ceramide. Ceramides can be produced or utilized through 3 main pathways; de novo biosynthesis, sphingomyelinase (SMase) pathway, or via the salvage pathway (Figure 4A). We observed increased levels of 3-phosphoglycerate (FC 1.56, *P* < 0.01) (Figure 4B), a key component to initiate the de novo sphingolipid synthesis, along with several intermediates including sphinganine (FC 1.41, *P* < 0.001) (Figure 4C) and sphinganine 1-phosphate (FC 1.35, *P* < 0.001) (Figure 4D).

In the salvage pathway, S1P was significantly increased (FC 1.24, *P* < 0.01) (Figure 4F), as well as hexadecasphingosine (d16:1) (FC 1.32, *P* < 0.01) (Figure 4E). S1P is cleaved into hexadecanal, a fatty aldehyde, and phosphoethanolamine; the latter also increased in CHM patients (FC 1.40, *P* < 0.01) (Figure 4G). Although hexadecanal was not detected in this study, its product, hexadecanoic acid (also called palmitic acid or palmitate), was not significantly altered in CHM patients (not shown).

A modest depletion of a number of sphingomyelins (SMs) was detected including SM(d18:1/20:0, d16:1/22:0) (FC 0.91, *P* < 0.05) (Figure 4H) and SM(d18:1/22:1, d16:1/24:1) (FC 0.92, *P* < 0.05) (Figure 4I). SMs are synthesized by the transfer of a phosphorylcholine residue from phosphatidylcholine (PC) to a ceramide by SM synthase. SMs can also be hydrolyzed back to release ceramides and phosphorylcholine residues by the action of SMase (20). In accordance, several PC were also detected in significantly lower levels in CHM patients, such as PC(16:0/22:6) (FC 0.88, *P* < 0.05) and PC(18:1/22:6) (FC 0.87, *P* < 0.05) (data not shown), implying phospholipid deregulation caused by REP1 deficiency.

Ceramide is considered the central molecule in the sphingolipid metabolic pathway. Surprisingly, none of the ceramides detected in this study showed significantly altered levels in CHM samples compared with controls (not shown). Overall, these results could suggest a compensatory mechanism, possibly mediated by REP2, to regulate the ceramide pool through an increase of both de novo and salvage pathways to possibly compensate for the underperformance of the SMase pathway.

### CHM patients exhibit broader alterations in lipid metabolism.

**Figure 5:**
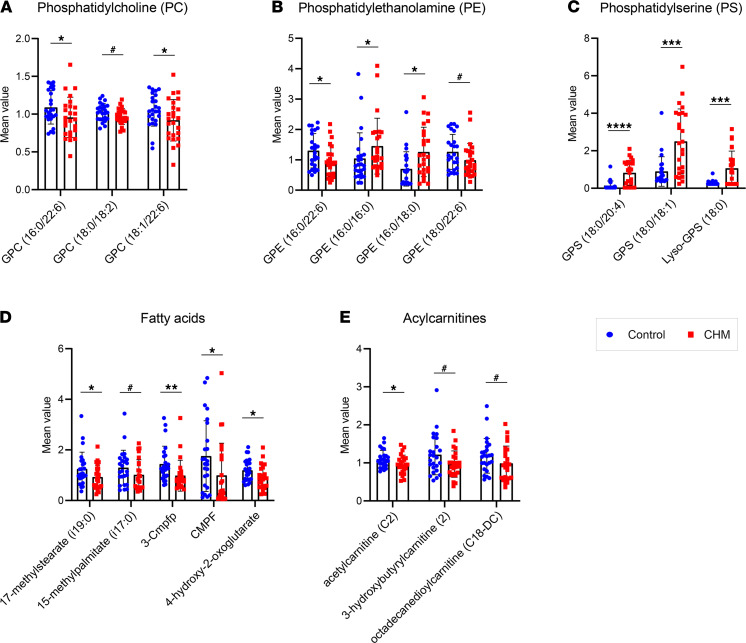
Metabolites involved in lipid metabolism subclasses differentially detected in CHM patients. (**A**–**E**) Bars represent mean ± SD of control (blue) and choroideremia (red) plasma samples (*n* = 25). *P* value was determined using matched pair *t* tests. *P ≤ 0.05, **P ≤ 0.01, ***P ≤ 0.001, ****P ≤ 0.0001. ^#^0.05 < *P* ≤ 0.1. GPC, glycerophosphocholine; GPE, gylcerophosphoethanolamine; GPS, glycerophosphoserine; 3-Cmpfp, 3-carboxy-4-methyl-5-pentyl-2-furanpropionate; CMPF, 3-carboxy-4-methyl-5-propyl-2-furanpropanoate.

Sphingolipid metabolism also contributes to glycerophospholipid metabolism, and disruption of this pathway can reduce glycerolipid levels, leading to broader lipid metabolism alterations. We observed differential effects of glycerolipid subclasses, with lower levels of PC intermediates (Figure 5A) but increased levels of phosphatidylethanolamine (PE) (Figure 5B) and PS lipids (Figure 5C). Of the latter group, 2 PS intermediates — 1-stearoyl-GPS(18:0) and 1-stearoyl-2-arachidonoyl-GPS(18:0/20:4) — were particularly increased in CHM patients, with nearly 4- and 6-FC compared with control samples, respectively (Figure 5C).

No major differences were found in CHM patients regarding long-chain FA levels, in contrast with the study from Zhang et al. (14). However, reduced levels of branched FAs 17-methylstearate (i19:0) (FC 0.75, *P* < 0.05) and 15-methylpalmitate (i17:0) (FC 0.78, *P* < 0.1) were observed, as well as several dicarboxylic FAs (DCFAs) (Figure 5D) and acylcarnitines (Figure 5E). Combined, these results point to impaired lipid oxidation in CHM patients.

### Human CHM lipid alterations are recapitulated in chm^ru848^ zebrafish.

**Figure 6:**
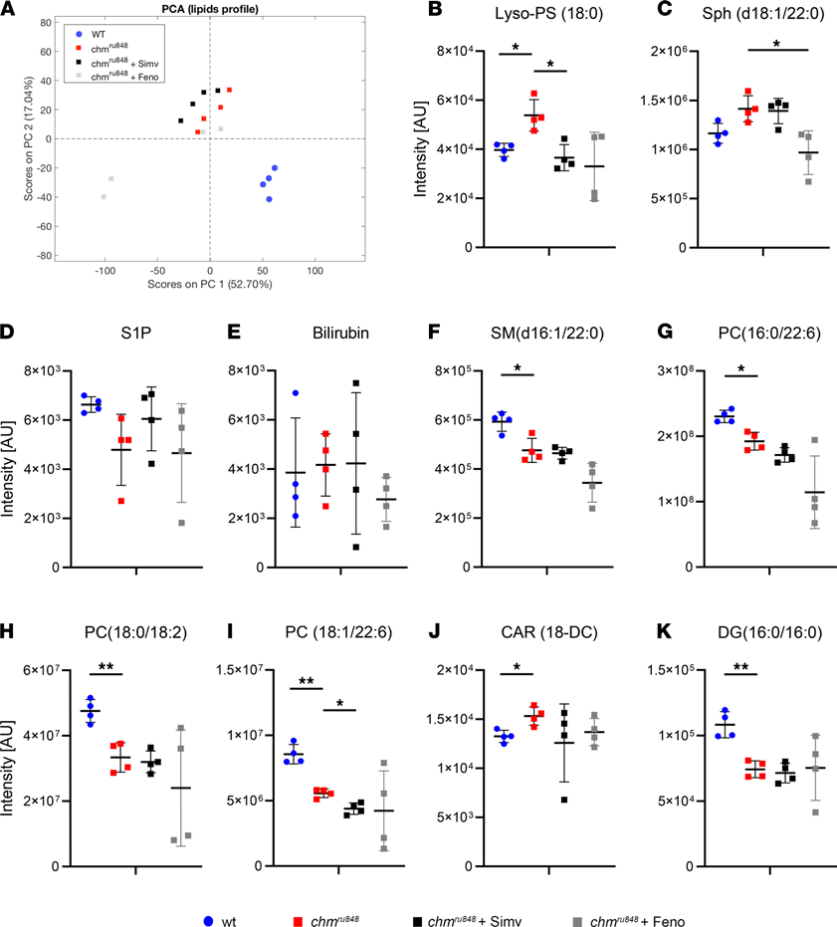
Lipidomic profiles of zebrafish. (**A**) PCA of day 6 *chm* *^ru848^* mutant fish untreated (red squares) or treated with 0.3 nM simvastatin (black squares) or 700 nM fenofibrate (gray squares), compared with WT fish (blue circles). (**B**–**K**) Scatter dot plots with key metabolites shared with human plasma metabolites and respective levels detected in all groups. Lines indicate mean ± SD (*n* = 4, 10 fish per group). *P* value was determined using 1-way ANOVA. *P ≤ 0.05, **P ≤ 0.01. a.u., arbitrary units; Lyso-PS, lysophosphoserine/1-stearoyl-GPS; Sph(d18:1/22:0), lactosyl-N-behenoyl-sphingosine; S1P, sphingosine-1-phosphate; CAR, carnitine; PC, phosphatidylcholine; SM, sphingomyelin.

LC-MS–based lipid profiling of *chm^ru848^* homozygous mutant zebrafish embryos at 6 dpf further corroborated the alterations in lipid metabolism detected in the plasma of CHM patients. Lipidomic-based PCA showed a clear separation between WT and *chm^ru848^* groups, which was not visible in the human analysis (Figure 6A). Twelve compounds were found with differential levels between *chm^ru848^* and WT samples that were also in the top 30 biochemicals from the human study, such as Lyso-PS (18:0) (1-stearoyl-GPS) and sphingosine (d18:1/22:0) (lactosyl-N-behenoyl-sphingosine). These metabolites were increased in both human and zebrafish CHM models (Figure 6, B and C). Metabolites found in significantly lower levels in *chm^ru848^,* as well as in human CHM samples, included SM(d16:1/22:0) (Figure 6F) and several PC compounds — i.e., PC(16:0/22:6), PC(18:0/18:2), and PC(18:1/22:6) (Figure 6, G–I).

S1P (Figure 6D), bilirubin (Figure 6E), SM(d16:1/24:1), and SM (d18.2/22:0) (not shown) were detected in *chm^ru848^*, but levels were not statistically significant compared with WT samples. In contrast, carnitine (C18-DC) (Figure 6J) and diacylglycerol (DG[16:0/16]) (Figure 6K) were significantly increased and decreased, respectively, in *chm^ru848^*, while the same compounds had opposite trends in the human study (0.05 < *P* ≤ 0.1) (Figure 5E, not shown). These differences are likely due to the presence of a single REP isoform in zebrafish, resulting in complete loss of REP activity, compared with humans, which have the compensatory action of REP2.

### Simvastatin versus fenofibrate treatment in chm^ru848^.

Since the lipid profile in both humans and zebrafish with CHM show disruption, the effect of HMG-CoA reductase inhibitors (also known as statins) and fibric acid derivatives (fibrates) were investigated using the zebrafish model. Statins have been shown to block the mevalonate pathway necessary for cholesterol synthesis, the same pathway necessary to produce isoprenoids essential for prenylation and REP1 function (9). Therefore, in an already compromised system, we wanted to investigate if fibrates (whose mode of action circumvents the mevalonate pathway) would be safer compounds to aid in normalizing lipid dysregulation without exacerbating the underlying biochemical genetic defect in CHM and potentially accelerating their retinal phenotype.

We administered predetermined doses of 0.3 nM simvastatin and 700 nM fenofibrate to WT and *chm^ru848^* mutant fish (*n* = 3, 50 embryos per group) from 24 hours, replenished daily till 9 dpf. There were no adverse effects seen in treated WT embryos, all demonstrating normal stereotyped motor behaviors that allowed them to navigate their environment, including slow and fast swimming bouts, with no signs of imbalance or lack of movement.

**Figure 7:**
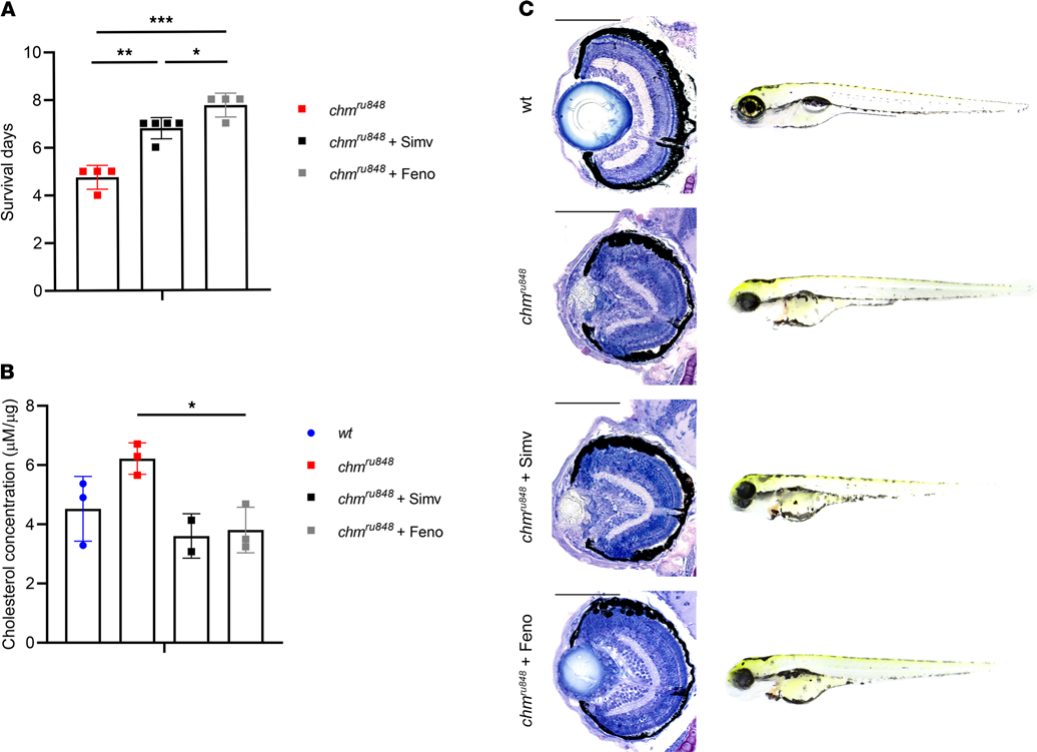
Characterization of *chm^ru848^* zebrafish treated daily with 0.3 nM simvastatin or 700 nM fenofibrate from 24 hours after fertilization. (**A**) Survival days of *chm^ru848^* fish untreated (red) or treated with simvastatin (black) or fenofibrate (gray) (*n* = 4, 50 fish per group). (**B**) Average levels of cholesterol (μM per μg of protein) in WT fish (blue circles), and *chm^ru848^* zebrafish untreated (red squares) or treated with simvastatin (black squares) or with fenofibrate (gray squares) at 6 dpf (*n* ≥ 2, 5 fish per condition). Data represent mean ± SD. (**C**) Retinal sections and wholemount morphology of WT, untreated *chm^ru848^* fish, and *chm^ru848^* fish treated with simvastatin or fenofibrate at 6dpf. Scale bar: 100 μm. *P* value was determined using 1-way ANOVA. *P ≤ 0.05, **P ≤ 0.01, ***P ≤ 0.001.

Characterization of untreated and treated *chm^ru848^* is presented in Figure 7. Survival studies show that untreated mutant zebrafish mean survival was 4.5 ± 0.5 days, while fish treated daily with simvastatin survived 6.8 ± 0.4 days and those with daily fenofibrate survived 7.8 ± 0.5 days (Figure 7A). Cholesterol levels were measured by Amplex Red Cholesterol Assay kit, which suggested a trend increase in mutant compared with WT fish (*P* = 0.032). Simvastatin- and fenofibrate-treated mutants both showed comparable reduction of cholesterol levels compared with untreated fish, although only the fenofibrate-treated group showed a significant reduction (*P* = 0.048) (Figure 7B). Histological analysis of *chm^ru848^* eyes at 6 dpf showed microphthalmia, cataract, and widespread retinal degeneration with loss of lamination and areas of RPE hypertrophy/atrophy (Figure 7C). Wholemount analysis of *chm^ru848^* embryos showed characteristic systemic defects, including pericardial and abdominal oedema, an uninflated swim bladder, and persistent yolk sac (Figure 7C) (21). Following treatment, no obvious phenotypic improvement was detected in simvastatin-treated retinas, while fenofibrate-treated mutants showed clearer retinal lamination and improved lens structure, although areas with significant RPE atrophy were still present (Figure 7C).

We then performed targeted lipidomic analysis of *chm^ru848^* mutant fish treated with 0.3 nM simvastatin or 700 nM fenofibrate, compared with untreated mutants and WT fish. PCA results show no clear distinction between treated groups and untreated *chm^ru848^* fish (Figure 6A).

The effect of simvastatin on *chm^ru848^* mutant fish leads to a decrease of Lyso-PS(18:0) (Figure 6B) and PC(18:1/22:6) (Figure 6I), but the remaining metabolites showed no significant changes between simvastatin-treated and untreated groups. Fibrates are PPARα agonist lipid-lowering drugs that seem to have a broader effect in lowering overall lipid levels compared with statins (22). Consistently, *chm* fish treated with 700 nM fenofibrate showed lower levels of most compounds selected in this lipidomics analysis compared with untreated *chm^ru848^* samples — metabolites that were already decreased in untreated mutant samples, particularly SMs (Figure 6F) and PCs (Figure 6, G–I), and were reduced even further. These compounds were largely unchanged by simvastatin, confirming the different modes of action between the drugs. However, it must be mentioned that there was a high variability in fenofibrate-treated *chm* zebrafish, suggesting that these results need further analysis or require a larger sample number to reduce variability and reach significance.

## Discussion

This is the first study to our knowledge to explore systemic disturbances in CHM through whole metabolomic analysis of blood plasma from 25 CHM patients and 25 age- and sex-matched controls using ultra high–performance liquid chromatography–tandem mass spectrometry (UPLC-MS/MS). Global analysis of the metabolomic data identified several biochemicals that can be adopted as biomarkers to distinguish between the 2 groups, including 1-steroyl-GPS and lactosyl-N-behenoyl-sphingosine. Pathway enrichment analysis highlighted significant alterations in CHM patients, the key pathways being lipid metabolism, particularly sphingolipid metabolism, cysteine and glutathione metabolism, tryptophan metabolism, and heme metabolism.

Sphingolipids are involved in different cellular processes and can have opposing effects; ceramides and sphinganines are considered proapoptotic and can mediate apoptosis, growth arrest, and senescence. In contrast, S1P is associated with cell proliferation, migration, and inflammation, and SMs are linked to cell growth and adhesion (20, 23). In the eye, oxidative stress is shown to increase ceramide and sphingosine levels, leading to photoreceptor death and RPE degradation, while S1P acts as a mediator of PR survival, preventing PR death during development and when exposed to oxidative stress (24). Analysis of sphingolipid metabolism revealed substantial imbalances in the presumptive ceramide pathway in CHM plasma, with increased levels of several sphinganines and sphingosines, including S1P, in parallel with lower levels of SMs, suggesting compromised ceramide production via the SMase pathway. It is unclear if these findings correlate with the retinal environment and disease severity, but measuring sphingolipid levels in PR and RPE derived from CHM patients, such as through generation of patient-derived induced pluripotent stem cells, may provide novel information on the effect of these metabolites in disease pathophysiology.

Sphingolipid metabolism perturbations can also suggest compromised degradation of the S1P pathway, which catalyzes the conversion of hexadecanal to hexadecanoic acid. Interestingly, this pattern is similar to metabolic perturbations identified in Sjögren-Larsson syndrome (SLS) (OMIM 270200), an autosomal-recessive, neurocutaneous disease characterized by ichthyosis, mental retardation, and spastic diplegia (25). SLS is caused by mutations in the *ALDH3A2* gene, which encodes the membrane-bound fatty aldehyde dehydrogenase (FALDH), which catalyzes the dehydrogenation of hexadecanal in the S1P degradation pathway (26). FALDH is present in the retina, RPE, and choroid (27), with ocular defects recently identified in SLS patients including perifoveal crystalline inclusions, RPE atrophy with lipofuscin granules, retinal thinning, and deficient macular pigment (28). Given the similar perturbations between diseases, we hypothesize that FALDH could also be a target for REP1, since it also has been found to require prenylation for proper localization and function (10). In fact, other aldehyde dehydrogenases, ALDH3B1 and ALDH3B2, are also reported to be prenylated, through both farnesylation and geranylgeranylation (29, 30).

Analysis of specific metabolites related to lipid metabolism pointed to disruption of different phospholipid classes. For example, while a reduction of PC intermediates in CHM patients was detected, we also observed increased levels of some PE and particularly high accumulation of PS lipids. These different phospholipid subclasses are metabolically and structurally similar; PS is synthesized by PS synthases 1 and 2 in the endoplasmic reticulum (ER), which exchange serine for choline or ethanolamine in PC or PE, respectively. Conversely, PS can be converted to PE by PS decarboxylase (PSD) in the mitochondria (31). These observations may imply decreased activity of these enzymes, particularly PSD. Increased PS levels have not been associated with any human phenotype to date, but PS are increased in neuronal cells through docosahexaenoic acid (DHA), inhibiting neuronal cell death (32). However, levels of DHA in CHM were not significantly altered (FC 0.72, *P* > 0.1). PS can also result from phospholipase A–type enzymatic activity; this is a massive enzymatic family involved not only in phospholipid remodeling, but also in cholesterol metabolism, cell differentiation, maintenance of mitochondrial integrity, cell proliferation, and cell death (33). Interestingly, some phospholipase A enzymes have also been described as essential for RPE survival and regulation of POS phagocytosis (34, 35), and they have been tightly linked to protein prenylation (36).

Unlike Zhang et al., no significant differences in saturated FAs, monounsaturated FAs, or polyunsaturated FAs were detected in our cohort (14), except for a reduction of branched FA 17-methylstearate (i19:0). These results were in accordance to the study by Radziwon et al., who did not detect FA metabolism differences in a cohort of 9 CHM patients (15). Nervonic acid, the only FA altered in CHM patients in both studies, was not detected through UPLC-MS. However, we observed reduced levels of a few DCFAs, as well as lower levels of acylcarnitines, which — combined with a trend reduction of branched FAs — may suggest impaired lipid β oxidation.

Through targeted lipidomic analysis, we show that the zebrafish CHM model, *chm^ru848^*, also presents distinct lipid profiles to the WT zebrafish. It should be noted that absence of rep1 in *chm^ru848^*, coupled with the evolutionary lack of a compensatory rep2 isoform in zebrafish, results in a severe systemic phenotype that leads to embryonic lethality by 5–6 dpf. This contrasts with the human form of the disease, and it may have a global influence on the metabolic parameters. However, systemic lipid abnormalities are characterized by overall decreased levels of several PC and SM, which are recapitulated in the CHM patient plasma. Accordingly, 1-steroyl-GPS is significantly increased in *chm^ru848^*, highlighting this compound as a putative biomarker for CHM, although its role and relation to CHM has not been uncovered yet.

Several recent studies have described the inhibitory effect of statins on the isoprenoid pathway, as well as on prenylation of several Rab proteins, namely Rab7 (10–12). The *chm^ru848^* zebrafish model showed a trend increase of cholesterol levels that were reduced following treatment with both statins and fibrates. Overall survival was increased in mutant fish treated with either drug; however, the fenofibrate-treated eyes showed a mild rescue with increased overall eye size and a less compacted lens. Interestingly, it was suggested that prenylation of GTP-binding proteins is also necessary for lens homeostasis (37). Coincidently, statin (lovastatin) treatment induced cataract formation in cultured rat lenses, which was alleviated by addition of GGPP (13), reinforcing the evidence that statins reduce the GGPP pool in the mevalonate pathway, making its use less indicated for CHM patients. We can also suggest that rep1 deficiency causes cataract formation in fish, likely due to deficient prenylation. Patients with CHM develop posterior subcapsular cataracts; however, the cause of this remains unclear. In retinitis pigmentosa (RP), increased aqueous flare (which is related to the amount of protein present from increased breakdown of the blood-retinal barrier and inflammation) has been found in patients with posterior subcapsular cataracts, potentially suggesting a similar inflammatory mechanism in CHM (38, 39). Considering the broad action of PPARα agonists, the mechanism by which fenofibrate treatment potentially reduces cataract formation in the *chm^ru848^* embryos is not fully understood, but it may be through lowering cholesterol levels, which can cause cataracts when disturbed (40). However, simvastatin-treated lenses showed no improvement, although overall cholesterol levels were also lower after treatment. It is therefore important to clarify the mechanism of action of fenofibrate in *chm^ru848^* zebrafish, since — considering the improvement of the ocular phenotype of mutant fish, as well as its overall increased survival and lower cholesterol levels — fenofibrate (and perhaps other PPARα agonists) could have some therapeutic potential for CHM.

In 2012, a phase 1/2 trial was initiated (NCT01654562; https://clinicaltrials.gov/ct2/show/NCT01654562) to examine the short-term effects of simvastatin on the vision of CHM male patients, evaluated by full-field scotopic threshold testing. The investigators hypothesized that they would see a reversible decrease in the dark-adapted vision in participants taking simvastatin; however, this study was terminated due to limited enrollment, with only 2 patients recruited. It is unlikely that, over a period of 5 weeks, a detectable change in full-field scotopic threshold testing or the other parameters, including microperimetry, would be a useful outcome metric. From this study, we would suggest a safer alternative for CHM patients would be to take fibrates to reduce cholesterol and overall lipid dysfunction. However, a trial of statin versus fibrate in those requiring treatment could be undertaken over a 12-month period measuring visual function parameters to assess for a decline, but numbers of patients would need to be high to achieve statistical significance in view of the intra- and interfamilial variability also seen with this disease.

Alterations in lipid catabolism are often linked to changes in oxidative stress. We observed mixed perturbations in the cysteine pathway that indicate altered demand for glutathione and may reflect a need to manage oxidative stress in CHM patients. While glutathione is typically not detected in plasma, loss of cysteine and associated dipeptide cysteinylglycine, as well as accumulation of hypotaurine are consistent with increased glutathione production. Additionally, differential changes in cysteinylglycine and 5-oxoproline support engagement of the glutathione cycle in CHM patients. This is consistent with our previous study showing elevated levels of oxidative stress (superoxide) in the retina of *chm^ru848^* zebrafish mutant embryos (41). Oxidative damage can lead to a number of chronic diseases such as atherosclerosis, cardiovascular diseases, stroke, diabetics, rheumatoid arthritis, cancer, aging, and other degenerative diseases in humans (42). Although we were unable to identify clear markers for these diseases in our study, exploring therapies focused on reducing oxidative stress levels may be beneficial in reducing any associated risk in CHM. Patient plasma revealed decreased levels of several known antioxidants; therefore, diet supplementation with antioxidant compounds like N-acetylcysteine (NAC) or even modulators of nuclear factor erythroid 2–related factor 2 (NRF2), the master regulator of antioxidant response, could be of interest.

Increased oxidative stress may also contribute to CHM ocular manifestations, since it was found to cause retinal PR death and RPE atrophy in RP, with a reduction in the reduced to oxidized glutathione ratio (GSH/GSSG) in aqueous humor (43). NAC was found to be an effective antioxidant in RP mouse models promoting cone survival and function (44), and a recent phase 1 clinical trial FIGUREHT-RP1 (NCT03999021 and NCT03063021) of orally administered NAC (maximum tolerated 1800 mg twice a day) showed improvement of both cone function and best-corrected visual acuity (BCVA) (45). As delivered orally, NAC may help reduce the oxidative stress in the retina and the plasma of CHM patients with wider systemic benefit than just halting or slowing further sight loss.

Tryptophan metabolism results in the synthesis of neurotransmitters serotonin and melatonin, and — via the kynurenine pathway — produces nicotinamide, which is linked to inflammation and neurotoxicity of the CNS. We further examined metabolic markers of inflammation; however, there was no strong evidence of involvement. Importantly, we observed a striking increase in serotonin levels in CHM patients. Serotonin regulates sleep, mood, and behavior and is also the precursor of melatonin, a powerful antioxidant essential for regulation of circadian rhythm (46). Serotonin is produced in the pineal gland and the gastrointestinal tract, but some can be produced in PR as a precursor of melatonin, whose production is defined by the levels of light captured by the retina. Furthermore, serotonin acts as a neuromodulator in retinal physiology and photoreceptor survival (47). Serotonin is catabolized by action of monoamine oxidase A (MAO-A) and is reuptaken by serotonin transporter (SERT); inhibitors of both enzymes increase serotonin levels and are used worldwide as antidepressants (47). Systemic high levels of serotonin can cause serotonin syndrome, characterized by anxiety, muscle tremors or spasms, rapid heartbeat, and high blood pressure (48). Mutations in MAO-A cause X-linked Brunner syndrome (OMIM 300615) which is characterized by increased monoamine levels like serotonin, dopamine, and norepinephrine and leads to mild mental retardation, aggressive behavior, sleep disorders, and mood swings (49). Although serotonin levels in these syndromes are difficult to compare with CHM, it would be important to elucidate the link with elevated serotonin, since there may be a subtle propensity for some of these features.

Serotonin can also regulate insulin secretion. Serotonylation is a posttranslational modification mechanism where transglutaminases add serotonin to the glutamine residues of GTPases, forming covalent bonds for activation of intracellular processes (50). Rab3a and Rab27, the latter a known target of REP1-dependent prenylation, are activated via this mechanism in the pancreas, which in turn promotes glucose-mediated insulin secretion (51). Interestingly, we observed significantly reduced levels of microbiome-related indoles, particularly indolepropionate, which has antioxidant properties and was recently associated with lower risk of developing type 2 diabetes mellitus (52). No correlation has been reported between CHM and diabetes, but these results suggest close monitoring of patients for insulin insufficiency.

Melatonin levels could not be detected in this study; however, it has recently been hypothesized as a potential antioxidant treatment for age-related macular degeneration (AMD), by reducing oxidative stress, inflammation, and apoptosis in the retina (53). AMD etiology has been compared with CHM, and recent metabolomic studies also revealed mitochondrial deficiency, as well as systemic carnitine and glutamine pathway defects (54–56). Furthermore, Laíns et al. showed decreased glycerophospholipids levels, particularly GPC, in AMD plasma samples (56). CHM and AMD may share a common metabolome; hence, the possible role of both serotonin and melatonin in the retina and RPE should be further elucidated, possibly opening new therapeutic avenues.

The cytochrome P450 superfamily is a key family of monooxygenase enzymes involved in metabolism of endogenous molecules, such as steroids and FA. Several of the metabolites that differed between CHM and control groups were connected to liver cytochrome activity, including reduced bilirubin and increased urobilin. Since heme levels were not significantly different, activity of heme oxygenase 1 (HO-1), a rate-limiting enzyme of heme catabolism, may have been impaired. Interestingly, HO-1 was found to be increased after mevalonate pathway inhibition using statins in mice macrophages; this change was dependent on prenylation, since addition of FPP or GGPP partially reversed this elevation (57).

Aside from the major metabolic perturbations discussed, there were other differentially identified metabolites of interest, such as ornithine, which was significantly increased (1.13-fold) in CHM. Ornithine is produced in the urea cycle by the splitting off of urea from arginine. Mutations in the ornithine aminotransferase gene (*OAT*) cause gyrate atrophy (GA) (OMIM 258870), which is characterized by increased ornithine serum levels and has a similar clinical phenotype to CHM, with patients presenting with night blindness and progressive chorioretinal atrophy, eventually leading to blindness (58). Ornithine is toxic to the RPE and retina; thus, lowering dietary intake can delay further retinal degeneration (59). No major systemic phenotypes are known to be associated with GA, but the increased ornithine levels in both disease groups suggest a close relationship between REP1 and OAT. Patients may benefit from dietary advice to reduce ornithine intake to prevent possible disease exacerbation.

Collectively, these results provide potentially novel insights into the systemic derangements in CHM that occur due to disruption of REP1 activity. CHM is unlikely to be an isolated retinal dystrophy due to the ubiquitous expression of REP1. To date, accumulation of unprenylated Rab proteins is the only disease mechanism described in CHM, but this study proposes putative enzymes, such as FALDH, cytochrome P450, or monoamine or heme oxygenases, that could be targets of systemic REP1. The metabolic perturbations must be considered as presymptomatic risk factors for more chronic systemic involvement. Further long-term natural history studies are required regarding CHM and aging to determine the prevalence of multisystemic manifestations. Therapeutic approaches could be developed for these modifiable risk factors, such as repurposing the S1P receptor functional antagonist, fingolimod, to counter the effects of S1P accumulation in CHM. Use of in vitro and in vivo CHM disease models will also prove fundamental to establish the connection between the compounds described herein and REP1 function, providing pathomechanisms in CHM, currently not completely understood.

## Methods

### Clinical evaluation.

Twenty-five unrelated patients under Moorfields Eye Hospital NHS Foundation Trust (London, United Kingdom) with clinically diagnosed CHM and molecularly confirmed *CHM* hemizygous mutations were included in this study, together with 25 age- and sex- matched controls. A detailed ocular and medical history was taken with comprehensive ophthalmic examination as part of routine care (Supplemental Table 1). ETDRS BCVA was measured. Patients with a clinical history of diabetes, hypercholesterolemia, or drug history of taking statins or any other medications were excluded.

### Assessment of dietary intake.

All CHM patients and control subjects were asked to complete a FFQ on their average consumption of various foods and drinks over the past 12 months. The validated FFQ comprised a list of 147 food items, and participants were asked to indicate their usual consumption from 1 of 9 frequency categories ranging from “never or less than once per month” to “6 or more times per day” (60). Individuals would have been excluded if their answers to > 10 items had been left blank, but this was not true for any of the participants. Nutrients were calculated using the UK Nutrient Database (61).

### Sample collection.

Blood plasma samples were collected from nonfasting CHM patients and age- and sex-matched healthy individuals (*n* = 25 per group), between 9 a.m. and 11 a.m. Plasma was extracted by centrifuging whole blood at 600*g* for 15 minutes at room temperature. Extracted plasma samples were aliquoted and stored at –80°C. Samples that had not previously been thawed were shipped on dry ice to Metabolon Inc.

### Metabolomics analysis.

Blood plasma metabolite extractions for UPLC-MS/MS were completed by Metabolon Inc., according to the protocol described in Supplemental Methods.

### Metabolic pathway networks and analysis.

To visualize and analyze small molecules within relevant networks of metabolic pathways, the detected metabolites in CHM patient and healthy control study groups were subjected to MetaboLync pathway analysis (MPA) software (www.portal.metabolon.com). Significantly altered pathways were determined by completing pathway set enrichment analysis within MPA software, which was determined by the following equation:

No. of significant metabolites in pathway (*k*)/total no. of detected metabolites in pathway (*m*)/total no. of significant metabolites (*n*)/total no. of detected metabolites (*N*). Thus, significantly altered pathways were determined by (*k*/*m*)/(*n*/*N*).

A pathway impact score greater than 1 indicated that the pathway contained a higher number of experimentally regulated compounds relative to the overall study in CHM patients relative to controls.

### Zebrafish husbandry.

The WT AB and *chm^ru848^* embryos were generated by natural pair-wise matings of identified heterozygous carriers. Embryos were raised at 28.5°C on a 14-hour light/10-hour dark cycle in a 90 mm petri dish containing aquarium water. The developmental stages are given in hours or days postfertilization (hpf/dpf), according to morphological criteria (62).

### Simvastatin and fenofibrate dosing of zebrafish.

For all the dosing, the drugs were prepared in aquarium water. The *chm^ru848^* mutant embryos were dechorionated at 10 hpf and treated at 24 hpf with either 0.3 nM simvastatin or 700 nM fenofibrate (63–65). The embryos were treated with a fresh dose of the drugs every 24 hours, and as a positive control, an equal number of *chm^ru848^* mutant embryos was kept in drug-free aquarium water. Survival of mutant larvae was recorded in days; *n* = 50 for each treatment group.

### Cholesterol assay.

Whole-body cholesterol was determined using the Amplex Red Cholesterol Assay kit (Invitrogen) according to the manufacturer’s instructions. Pools of 5 WT AB and *chm^ru848^* embryos per condition were collected and homogenized in sample buffer on ice. Cholesterol concentrations were measured using a TECAN microplate spectrofluorometer with an excitation wavelength of 545 nm and an emission wavelength of 590 nm. Concentrations were quantified using authentic cholesterol standards (provided in the kit) and estimated based on a gradient dilution of the cholesterol standards.

### Retinal histology and wholemount morphology.

Retinal and wholemount morphology analyses were performed as previously described (21). All images were edited using ImageJ (NIH).

### Lipidomic analysis of zebrafish.

Ten zebrafish were pooled for each sample (with 4 biological samples in total). Homogenization to smooth emulsion was achieved by sonication of each pool in 100 μL water. Liquid-liquid extraction of this emulsion was performed similar to Izzi-Engbeaya et al. (66). In brief, the homogenized pool was mixed with isopropanol (IPA) spiked with internal standards 1:4 (v/v) in a microcentrifuge tube, incubated at 4°C with shaking at 1400 rpm for 2 hours, followed by centrifugation for 10 minutes at 3680*g* at 4°C, and the supernatant was used for injection. LC-MS data were acquired as previously described (66). Feature extraction from LC-MS lipid positive and negative ion modes spectra was performed in XCMS (67) and by in-house scripts.

Lipid annotation was achieved by MS/MS acquisition, followed by matching to in-house and online databases. Measurement of predefined lipid of interests were detected, integrated, and reported using an in-house open source package (https://doi.org/10.5281/zenodo.3523406).

### Statistics.

Mann-Whitney *U* tests were used to compare age and dietary variables between patients and controls. Metabolite profiles in CHM patients and controls were quantified in terms of relative abundance and median scaled to 1. Following log transformation and imputation of missing values, if any, with the minimum observed value for each compound imputed, statistical analyses were performed using ArrayStudio (Omicsoft) or R version 2.14.2 (https://www.r-project.org/). Metabolite profile distinctions between CHM patients and healthy individuals were evaluated by matched pair *t* tests. An estimate of the FDR (*q* value) was calculated, and a threshold of *q* ≤ 0.10 was used to correct for false discovery of statistically significant compounds. FC was determined by dividing the relative abundance of each metabolite in the CHM patients blood plasma by the relative abundance of the metabolite in the blood plasma of healthy control individuals. FC values with *P* ≤ 0.05 and *q* ≤ 0.10 were considered statistically significant, while FC values with 0.05 < *P* < 0.10 were considered as trending toward significance.

For zebrafish survival and cholesterol measurements, significance was calculated by 1-way ANOVA. For lipidomic analysis, means and SDs were calculated using 10 fish per group (*n* = 4). Statistical analysis was performed by 1-way ANOVA using GraphPad Prism 8 v8.4.2 (GraphPad software; https://www.graphpad.com/).

Multivariate statistical analysis for lipidomic profiling of zebrafish (i.e., PCA) was based upon the XCMS data sets from LC-MS spectra of zebrafish extracts and was performed using MATLAB based PLS_Toolbox version 8.7.1 (2019) (Eigenvector Research Inc.; http://www.eigenvector.com).

### Study approval.

The study protocol adhered to the tenets of the Declaration of Helsinki and received approval from Moorfields Eye Hospital NHS Foundation Trust and the National Research Ethics Committee (REC12/LO/0141). Written informed consent was obtained from all participants prior to their inclusion in this study.

Zebrafish (WT AB and *chm^ru848^*) were bred and maintained in the University College London animal facility according to standard protocols and the guidelines of the ARVO Statement for the Use of Animals in Ophthalmic and Vision Research (68).

## Author contributions

DLC analyzed the human and zebrafish data, performed statistical analysis, and wrote the original draft; RR collected samples and wrote the first draft; DTW collected samples, performed zebrafish experiments, and analyzed data; AM and AA performed clinical evaluation of patients; VHVDS and PT performed target lipidomics and preliminary data analysis; NO contributed to data analysis; JS and AAW conducted nutritional assessment of all participants; MM conducted the study, analyzed data, acquired funding, and wrote the manuscript. All authors reviewed and approved the manuscript before submission.

## Supplementary Material

Supplemental data

## Version 1 03/23/2021

In-Press Preview

## Version 2 05/10/2021

Electronic publication
