# Supplementary material for: REP1 deficiency causes systemic dysfunction of lipid metabolism and oxidative stress in choroideremia
Source: JCI Insight. 2021 May 10;6(9):e146934. doi: 10.1172/jci.insight.146934 (PMC8262314; doi:10.1172/jci.insight.146934)
Supplement: Supplemental data [file jciinsight-6-146934-s120.pdf]

# Supplementary Materials

## MATERIALS AND METHODS

### HUMAN PLASMA METABOLOMICS ANALYSIS

Each sample was accessioned into the Metabolon Laboratory Information Management System (LIMS) and assigned a unique identifier associated with the original de-identified study code number. The identifier was used to track all sample handling, tasks and results.

Samples were prepared using the automated MicroLab STAR® system from Hamilton Company (Reno, NV, USA). Several recovery standards were added prior to the first step in the extraction process for quality control purposes. To remove protein, dissociate small molecules bound to protein or trapped in the precipitated protein matrix, and to recover chemically diverse metabolites, proteins were precipitated with methanol under vigorous shaking for 2 min (Glen Mills GenoGrinder 2000) followed by centrifugation. The resulting extract was divided into five fractions: two for analysis by two separate reverse phase (RP)/UPLC-MS/MS methods with positive ion mode electrospray ionization (ESI), one for analysis by RP/UPLC-MS/MS with negative ion mode ESI, one for analysis by HILIC/UPLC-MS/MS with negative ion mode ESI, and one sample was reserved for backup. Samples were placed briefly on a TurboVap® (Zymark) to remove the organic solvent. The sample extracts were stored overnight under nitrogen before preparation for UPLC-MS/MS analysis.

The UPLC-MS/MS platform utilised a Waters ACQUITY ultra-performance liquid chromatography (UPLC) and a Thermo Scientific Q-Exactive high resolution/accurate mass spectrometer interfaced with a heated electrospray ionization (HESI-II) source and Orbitrap mass analyzer operated at 35,000 mass resolution. The dried sample extract was reconstituted in acidic or basic UPLC-compatible solvents, each of which contained 11 to 13 injection standards at fixed concentrations (14). Two aliquots were analysed using acidic positive ion conditions, chromatographically optimised for hydrophilic or hydrophobic compounds and the other using basic negative ion optimised conditions, using separate dedicated columns (Waters UPLC BEH C18-2.1x100 mm, 1.7 µm). Extracts reconstituted in acidic conditions were gradient eluted using water and methanol, containing 0.05% perfluoropentanoic acid (PFPA) and 0.1% formic acid (FA) for hydrophilic compounds or water, methanol, acetonitrile, 0.05% PFPA and 0.01% FA for hydrophobic compounds. Basic extracts were also gradient eluted using methanol and water, but with the addition of 6.5mM Ammonium Bicarbonate at pH 8. The fourth aliquot was analysed via negative ionization following elution from a HILIC column (Waters UPLC BEH Amide 2.1x150 mm, 1.7 µm) using a gradient consisting of water and acetonitrile with 10 mM Ammonium Formate, pH 10.8. The MS analysis alternated between MS and data-dependent MS/MS scans using dynamic exclusion and the scan range covered 70-1000 m/z.

Raw data was extracted and peak-identified as per Metabolon's hardware and software. Biochemical identifications were based on three criteria: retention index within a narrow RI window of the proposed identification, accurate mass match to the library +/- 10 ppm, and the MS/MS forward and reverse scores between

the experimental data and authentic standards. The MS/MS scores were based on a comparison of the ions present in the experimental spectrum to the ions present in the library spectrum. More than 3300 commercially available purified standard compounds have been acquired and registered into LIMS for analysis on all platforms for determination of their analytical characteristics (REF).

**Supplementary Table 1.** Clinical description of CHM patients included in this study.

| ID | DOB | Visual acuity OD | Visual acuity OS | FA_OD (mm2) | FA_OS (mm2) | Retinal Thickness OD (µm) | Retinal Thickness OS (µm) | Choroidal Thickness OD (µm) | Choroidal Thickness OS (µm) | EZ OD (µm) | EZ OS (µm) | CHM variant (NM_000390.4) |
|---|---|---|---|---|---|---|---|---|---|---|---|---|
| P1 | 1986 | 6_6 | 6_6 | 17.23 | 21.03 | 259 | 258 | 229 | 278 | 4075 | 4503 | c.116+1G>A |
| P2 | 1983 | 6_6 | 6_6 | 7.95 | 7.17 | 335 | 346 | 141 | 148 | 2521 | 1951 | c.759delA; p.(Tyr254Metfs*37) |
| P3 | 1979 | 6_5 | 6_5 | 0 | 0 | 221 | 245 | 252 | 263 | 5059 | 5234 | c.-98C>T |
| P4 | 1980 | 6_6 | 6_6 | 28.1 | 32.02 | 312 | 254 | 254 | 224 | 4826 | 3684 | c.116+1G>A |
| P5 | 1996 | 6_9 | 6_12 | 16.3 | 14.87 | 276 | 299 | 374 | 373 | 3950 | 2830 | Deletion exon 3-intron 4 |
| P6 | 1959 | HM | 6_15 | 4.74 | 0.99 | 80 | 241 | 76 | 83 | 259 | 647 | c.1347C>G; p.(Tyr449*) |
| P7 | 1987 | 6_7.5 | 6-9.5 | 16.24 | 20.29 | 268 | 243 | 219 | 233 | 3308 | 3824 | c.808C>T; p.(Arg270*) |
| P8 | 1989 | 6_6 | 6_6 | 35.2 | 33.08 | 246 | 257 | 286 | 282 | 4600 | 4781 | c.808C>T; p.(Arg270*) |
| P9 | 1989 | 6_7.5 | 6_7.5 | 2.26 | 1.69 | 227 | 237 | 138 | 168 | 1567 | 2127 | c.126C>G; p.(Tyr42*) |
| P10 | 1963 | 6_9 | 6_12 | 22.41 | 4.25 | 320 | 327 | 200 | 138 | 3597 | 2053 | Deletion exons 3-15 |
| P11 | 1955 | 6_5 | 6_19 | 9.32 | 3.86 | 294 | 240 | 225 | 148 | 2716 | 729 | c.715C>T; p.(Arg239*) |
| P12 | 1965 | LP | 6_24 | 0 | 0 | 156 | 230 | 93 | 149 | 3047 | 828 | c.495dupT; p.(Ala166Cysfs*8) |

| | | | | | | | | | | | | |
|---|---|---|---|---|---|---|---|---|---|---|---|---|
| P13 | 1974 | 6_7.5 | 6_4.8 | 11.34 | 19.65 | 334 | 350 | 131 | 135 | 2455 | 2900 | c.877C>T p.Arg293* |
| P14 | 1967 | 6_15 | LP | 0.41 | 0.16 | 221 | 157 | 114 | 60 | 716 | 400 | c.698C>G; p.(Ser233*) |
| P15 | 1972 | 6_9.5 | HM | 1.99 | 0.5 | 238 | 190 | 166 | 140 | 633 | 0 | Deletion exons 3-15 |
| P16 | 1970 | 6_9 | 6_6 | 7.68 | 5.97 | 221 | 246 | 127 | 133 | 1177 | 740 | c.649_652delTACT; p.(Tyr217Hisfs*14) |
| P17 | 1987 | 6_6 | 6_6 | 9.57 | 8.04 | 308 | 339 | 193 | 168 | 2969 | 2597 | c.1245_1246delins14 |
| P18 | 1976 | 6_6 | 6_4.8 | 20.65 | 22.88 | 255 | 263 | 193 | 210 | 1804 | 4020 | c.715C>T; p.(Arg239*) |
| P19 | 1989 | 6_6 | 6_6 | 22.61 | 24.65 | 213 | 239 | 181 | 152 | 3090 | 2056 | c.715C>T; p.(Arg239*) |
| P20 | 1997 | 6_12 | 6_12 | 18.73 | 14.39 | 268 | 281 | 230 | 243 | 3817 | 3542 | c.877C>T p.(Arg293*) |
| P21 | 1994 | 6_9 | 6_9 | 11.76 | 12.14 | 272 | 268 | 244 | 213 | 2762 | 2174 | Deletion exons 1-11 |
| P22 | 1955 | 6_48 | 6_12 | 17.94 | 12.84 | 196 | 424 | 109 | 184 | 1824 | 3023 | c.715C>T; p.(Arg239*) |
| P23 | 1980 | 6_9 | 6_9 | 30.79 | 38.27 | 291 | 292 | 248 | 211 | 5969 | 4979 | Deletion exons 1-15 |
| P24 | 1963 | LP | HM | 0 | 0 | 163 | 178 | 136 | 145 | 0 | 0 | Deletion exons 10-11 |
| P25 | 1968 | 6_6 | 6_6 | 17.44 | 12.46 | 231 | 275 | 141 | 145 | 2116 | 1356 | c.799C>T; p.(Arg267*) |

**Abbreviations:** DOB, date of birth; FA, fluorescein angiography; OD, right eye; OS, left eye;; EZ, ellipsoid zone; LP, light perception; HM, hand movement.

**Supplementary Table 2.** Dietary intake of CHM patients and controls.

| | **Mean values Control** | **Mean values CHM** | ***p*-value** |
|---|---|---|---|
| Fat (g) | 78.2 | 80.1 | 0.635 |
| Energy (Kcal) | 2091 | 2183 | 0.541 |
| Protein (g) | 89.7 | 91.5 | 0.621 |
| NSP (g) | 17.7 | 19.6 | 0.357 |
| % energy from fats | 33.3 | 32.9 | 0.977 |
| % energy from saturated fatty acids | 12.4 | 12.0 | 0.516 |
| % energy from polyunsaturated fatty acids | 5.5 | 5.6 | 0.541 |
| % energy from monounsaturated fatty acids | 11.8 | 11.7 | 0.869 |
| Carotene (ug) | 3877 | 4623 | 0.399 |
| Vitamin C (mg) | 132.2 | 140.5 | 0.528 |
| Fruits (portions/day) | 2.14 | 2.19 | 0.712 |
| Vegetables (portions/day) | 4.31 | 4.50 | 0.607 |
| Leafy vegetables (portions/day) | 0.85 | 1.13 | 0.264 |
| Meat (portions/day) | 1.11 | 1.23 | 0.541 |
| Fish (portions/day) | 0.36 | 0.42 | 0.565 |
| Milk (pints/day) | 0.57 | 0.47 | 0.968 |
| Other dairy (portions/day) | 0.87 | 0.89 | 0.46 |
| Cereal foods (portions/day) | 5.19 | 5.46 | 0.93 |

**Supplementary Table 3.** Top 30 metabolites with most separation potential between CHM and control groups. Figures in coloured boxes represent fold change (FC) values significantly increased (red) or decreased (green) in choroideremia patients compared to age-matched controls.

| Metabolite | Sub Pathway | Mean Control | Mean CHM | CHM/ Control FC | *p* value |
|---|---|---|---|---|---|
| Sphingadienine | Sphingolipid Metabolism | 0.8736 | 1.4326 | 1.64 | 3..6471E-06 |
| Iminodiacetate (IDA) | Chemical | 1.5682 | 0.9047 | 0.58 | 1.9034E-08 |
| Cysteine S-sulphate | Methionine, Cysteine, SAM and Taurine Metabolism | 8.2307 | 0.7902 | 0.10 | 2.7906E-10 |
| Cysteine | Methionine, Cysteine, SAM and Taurine Metabolism | 1.0930 | 0.8869 | 0.81 | 6.8246E-7 |
| Sphinganine | Sphingolipid Metabolism | 0.8402 | 1.1818 | 1.41 | 3.2126E-07 |
| Cysteinylglycine | Glutathione Metabolism | 1.6662 | 0.9251 | 0.56 | 5.9919E-06 |
| Sarcosine | Glycine, Serine and Threonine Metabolism | 1.1676 | 0.8454 | 0.72 | 3.8408E-06 |
| 1-stearoyl-GPS (18:0)* | Lysophospholipid | 0.2791 | 1.0650 | 3.82 | 7.794E-05 |
| Adenosine 5' monophosphate (AMP) | Purine Metabolism | 0.8544 | 1.4386 | 1.68 | 0.0003 |
| 1-stearoyl-2-arachidonoyl-GPS (18:0/20:4) | Phosphatidylserine | 0.1410 | 0.8234 | 5.84 | 2.274E-5 |
| Phosphoethanolamine | Phospholipid Metabolism | 0.9025 | 1.2660 | 1.40 | 0.0012 |
| 1-stearoyl-2-oleoyl-GPS (18:0/18:1) | Phosphatidylserine | 0.8936 | 2.4976 | 2.79 | 0.0001 |
| Aspartate | Alanine and aspartate Metabolism | 0.8983 | 1.1075 | 1.23 | 0.0021 |
| Cortisone | Corticosteroids | 1.1314 | 0.8424 | 0.74 | 0.0201 |
| Cys-gly oxidized | Glutathione Metabolism | 0.8730 | 1.1796 | 1.35 | 0.0201 |
| Serotonin | Tryptophan Metabolism | 0.4484 | 1.7132 | 3.82 | 0.0082 |
| Hypotaurine | Methionine, Cysteine, SAM and Taurine Metabolism | 0.8833 | 1.3528 | 1.53 | 0.0001 |
| 3-phosphoglycerate | Glycolysis, Gluconeogenesis, and Pyruvate Metabolism | 0.8156 | 1.2728 | 1.56 | 0.0018 |
| Hexadecasphingosine (d16:1)* | Sphingolipid Metabolism | 0.7790 | 1.0263 | 1.32 | 0.0043 |

| | | | | | |
|---|---|---|---|---|---|
| Phenylacetate | Phenylalanine Metabolism | 0.6640 | 1.4180 | 2.14 | 0.0053 |
| 3-methylcytidine | Pyrimidine Metabolism | 1.1051 | 0.8807 | 0.80 | 0.0026 |
| Bilirubin (Z,Z) | Hemoglobin and Porphyrin Metabolism | 1.2231 | 0.9971 | 0.82 | 0.0053 |
| Sphinganine 1-phosphate | Sphingolipid Metabolism | 0.8836 | 1.1957 | 1.35 | 0.0007 |
| Lactosyl-N-behenoyl-sphingosine (d18:1/22:0)* | Sphingolipid Metabolism | 0.8854 | 1.0297 | 1.16 | 0.0078 |
| Sphingosine 1-phosphate | Sphingolipid Metabolism | 0.9226 | 1.1452 | 1.24 | 0.0156 |
| 3-(3-hydroxyphenyl) propionate | Benzoate Metabolism | 1.7713 | 0.7427 | 0.42 | 0.0006 |
| 3-carboxy-4-methyl-5-propyl-2-furan propanoate (CMPF) | Fatty Acid, Dicarboxylate | 1.7559 | 1.2924 | 0.74 | 0.0078 |
| N-acetyl-2-aminooctanoate* | Fatty acid, Amino | 1.5519 | 0.9568 | 0.62 | 0.0022 |
| Beta-cryptoxanthin | Vitamin A Metabolism | 1.6441 | 1.1354 | 0.69 | 0.003 |
| Inosine 5’-monophosphate (IMP) | Purine Metabolism | 0.5003 | 1.2011 | 2.40 | 0.0222 |

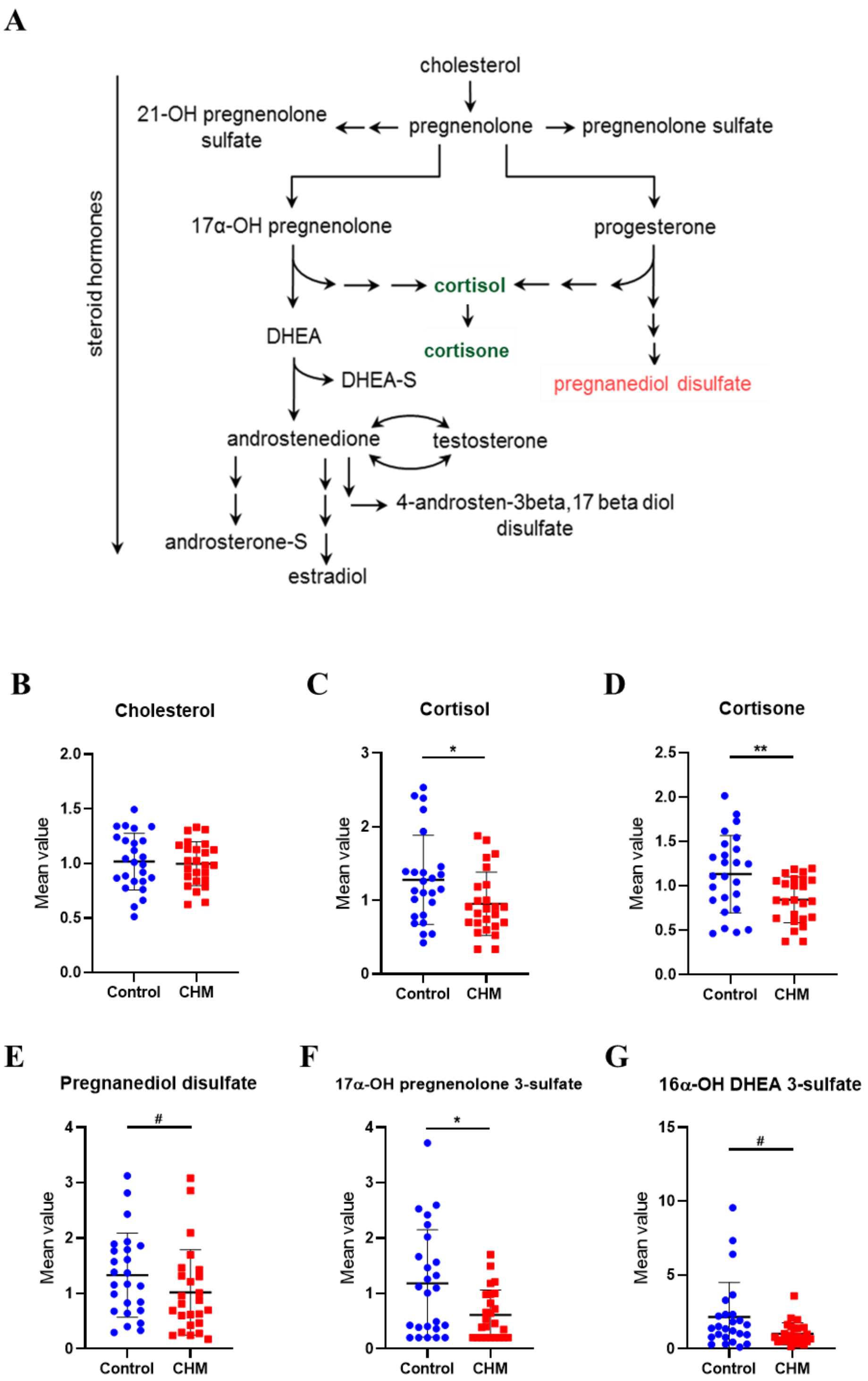


**Supplementary Figure 1. Biochemicals from steroids metabolism pathway altered in CHM patients.** (A) Schematics of steroid metabolism pathway. B-G) Selected metabolites altered in CHM vs control. Levels in CHM patient samples are represented in red and control samples in blue Values are shown in scatter dot plots with lines indicating mean ± SD (n=25). *p* value was determined using matched pair *t* tests: $^{\#}$ $0.05 < p \leq 0.1$, * $p \leq 0.05$, ** $p \leq 0.01$.
